# Screening of Obstructive Sleep Apnea Syndrome by Electronic-Nose Analysis of Volatile Organic Compounds

**DOI:** 10.1038/s41598-017-12108-w

**Published:** 2017-09-20

**Authors:** Simone Scarlata, Giorgio Pennazza, Marco Santonico, Simona Santangelo, Isaura Rossi Bartoli, Chiara Rivera, Chiara Vernile, Antonio De Vincentis, Raffaele Antonelli Incalzi

**Affiliations:** 1 Geriatrics, Department of Respiratory Pathophysiology, Campus Bio-Medico University and Teaching Hospital, Rome, Italy; 2 Centre for Integrated Research - CIR, Department of Electronics for Sensor Systems, Campus Bio-Medico University, Rome, Italy; 3 Department of Hepatology, Chair of Internal Medicine, Campus Bio-Medico University and Teaching Hospital, Rome, Italy

## Abstract

Obstructive Sleep Apnea Syndrome (OSAS) carries important social and economic implications. Once the suspicion of OSAS has arisen, Polysomnography (PSG) represents the diagnostic gold standard. However, about 45% of people who have undergone PSG are free from OSAS. Thus, efforts should be made to improve the selection of subjects. We verified whether the pattern of Volatile Organic Compounds (VOCs) helps to select patients amenable to PSG. We studied 136 subjects (20 obese non-OSAS, 20 hypoxic OSAS, 20 non-hypoxic OSAS, and 20 non-hypoxic Chronic Obstructive Pulmonary Disease (COPD) vs 56 healthy controls) without any criteria of exclusion for comorbidity to deal with a real-life population. VOCs patterns were analyzed using electronic-nose (e-nose) technology. A Discriminant Analysis (Partial Least Square-Discriminant Analysis) was performed to predict respiratory functions and PSG parameters. E-nose distinguished controls (100% correct classification) from others and identified 60% of hypoxic, and 35% of non-hypoxic OSAS patients. Similarly, it identified 60% of COPD patients. One-by-one group comparison yielded optimal discrimination of OSAS vs controls and of COPD vs controls (100% correct classification). In conclusion, e-nose technology applied to breath-analysis can discriminate non-respiratory from respiratory diseased populations in real-life multimorbid populations and exclude OSAS. If confirmed, this evidence may become pivotal for screening purposes.

## Introduction

Obstructive Sleep Apnea Syndrome (OSAS) is a highly prevalent condition with important social and economic implications^1^. In fact, OSAS is associated with cardiovascular, neurological and metabolic problems as well as with car accidents^2–6^. Most of these problems are fully or largely preventable with nocturnal ventilation through Continuous Positive Airway Pressure (C-PAP)^7^, at least in the symptomatic patient, since the benefit of treatment in the absence of symptoms is currently debated^8^. Thus, it is mandatory to recognize and treat OSAS patients in a timely manner. Once clinical suspicion of OSAS has arisen, Polysomnography (PSG) represents the gold standard diagnostic test, but it is cost and time consuming and requires dedicated personnel and setting. Furthermore, up to 45% of people who have undergone PSG are actually free from OSAS^9^. Thus, efforts should be made to improve the selection of subjects for PSG. Unfortunately, dedicated questionnaires and existing screening instruments could meet this goal only to some extent^10–12^. Furthermore, aging accounts for changing phenotype and symptoms of OSAS patients: in aged patients, obesity is less prevalent than in young and adult OSAS patients, and the same is true for snoring, whereas nocturia, an important effect of OSAS, may be misattributed to urological problems^13,14^. This makes the selection for PSG more and more difficult as the average age of the screened population increases. OSAS prevalence is strictly age-related, reaching 15–20% after the age of 70^15^, although some authors indicate that it may slightly decrease in the oldest patients^16^. Thus, in this population, the development of a reliable, inexpensive and simple screening instrument would be highly desirable. E-nose technology seems promising in this regard. It is a non-invasive and inexpensive technique oriented to Volatile Organic Compounds (VOCs) profiling in the exhaled breath for diagnostic and prognostic purposes. Indeed, profiling the composition of the exhaled breath, i.e. the pattern of VOCs, through e-nose is a simple, non invasive, rapid and inexpensive procedure. E-nose has been proved to have notable discriminative properties among different respiratory diseases, such as lung tumors, Chronic Obstructive Pulmonary Disease (COPD) and asthma^17–19^. Breath print (BP) analysis might complement or substitute for the questionnaire in the screening with the aim of improving PSG cost/effectiveness^11^. Furthermore, at least in people free from important comorbidity, BP might be used to monitor the response to and the compliance with CPAP. Finally, analysis of BP might shed light on the heterogeneity of OSAS phenotypes^19^.

To our knowledge, it has been tested in OSAS patients only on few occasions^19–24^. In all these studies, VOC patterns very reliably distinguished OSAS from normal subjects, and in two studies, the patterns dramatically changed in OSAS patients either after a single night or after three months of C-PAP therapy^19–22^. Interestingly, findings from Greulich *et al*. in 2013 suggested that e-nose might be used to screen subjects more likely to be diagnosed to have OSAS by PSG^22^; however, the study compares OSAS patients with healthy subjects, whereas in real life the differential diagnosis could vary depending on the clinical context^20^. Dragonieri *et al*. found that VOCs of OSAS and non-OSAS obese subjects largely overlap, and hypothesized that the systemic inflammation accounts for these analogies^23^. OSAS patients studied by Greulich had a very low prevalence of comorbidity known to influence BP^22^. Indeed, highly prevalent comorbidities, especially those characterized by a remarkable systemic inflammation, such as COPD and diabetes, frequently coexist with OSAS^25^ and their impact on VOCs profile is not completely ascertained.

Both COPD and diabetes are known to promote tissue oxidative stress, bloodstream inflammatory cytokine release and systemic inflammation. It is therefore likely that this may impact the VOCs composition^26,27^ and make the interpretation of VOCs profiles in OSAS more challenging.

The aim of this study is to verify to which extent e-nose technology can effectively distinguish OSAS people from healthy controls, as well as from non-OSAS obese and COPD patients. Assessing the discriminatory power of e-nose in real life conditions would allow for testing the potential usefulness of e-nose as a pre-PSG screening procedure.

## Methods

The study was approved by the Campus Bio Medico University Ethical Committee (Protocol no. 47/11 ComEt CBM, Rome, 12/05/2011). All patients signed an informed consent form in order to participate in the study. All methods were performed in accordance with the relevant guidelines and regulations.

### Participants

Eighty subjects (20 severely obese non-OSAS; 20 hypoxemic and severely obese OSAS; 20 non hypoxemic, mildly obese, OSAS; and 20 non hypoxemic, non obese COPD in stage A according to the Global initiative for COPD recommendations, GOLD) attending the outpatient clinic for respiratory diseases at Campus Bio-Medico Teaching Hospital were recruited (see Table 1).

**Table 1:** Anthropometric and demographic characteristics of the study groups.

	Controls (n = 56)	Obese non-OSAS (n = 20)	Non-hypoxic OSAS (n = 20)	Hypoxic OSAS (n = 20)	COPD (n = 20)	p-value
Age mean (SD)	66.8 (11.0)	59.7 (13.2)	62.7 (13.0)	64.0 (12.9)	64.5 (9.4)	0.55
Males n° (%)	35 (62.5)	12 (60.0)	12 (60.0)	14 (70.0)	14 (70.0)	0.77
BMI mean (SD)	25.6 (3.4)	35.0 (3.7)	30.3 (5.1)	36.7 (6.0)	25.1 (3.3)	0.003
T90 mean (SD)	1.2 (0.5)	3.4 (4.0)	4.8 (3.8)	48.8 (19.7)	14.3 (6.7)	<0.001
Current Smokers n° (%)	8 (14.0)	5 (25)	3 (15)	8 (40)	0	0.038
Number of Comorbidities mean (SD)	0.62 (0.6)	1.8 (1.7)	1.9 (1.7)	1.5 (1.5)	1.0 (1.2)	0.61
Hypertension n° (%)	31 (55.1)	10 (50)	14 (70)	13 (65)	5 (25)	0.048
Diabetes Mellitus n° (%)	4 (7.0)	6 (30)	5 (25)	7 (35)	1 (5)	0.73
Chronic Heart Failure n° (%)	0	2 (10)	0	3 (15)	3 (15)	0.07
Renal Failure n° (%)	0	1 (5)	0	1 (5)	0	0.56
Atrial Fibrillation n° (%)	0	3 (15)	2 (10)	3 (15)	1 (5)	0.67

Abbreviations: OSAS = Obstructive Sleep Apnea Syndrome; COPD = Chronic Obstructive Pulmonary Disease; BMI = Body Mass Index; T90 = Time with Oxygen Saturation below 90%.

The COPD group consisted of non-obese subjects. The fifty-six healthy, non-obese controls, free from both COPD and OSAS, were recruited among people attending the outpatient surgical department of the same hospital for minor surgical procedures. The group of obese, non-OSAS subjects included subjects with clinical features consistent with OSAS, but with a negative PSG. The clinical suspicion of OSAS was based on the presence of any of the following known clinically predictive symptoms and signs: snoring, abnormal sleep duration and schedule, daytime nap habits, excessive daytime sleepiness, increased neck circumference, morbid obesity (BMI > 36), facial or oropharyngeal dysmorphisms (macroglossia, micrognathism, uvulopalatal hypertrophy, or velopharinx collapse), otherwise unexplained nocturia, sleep apneas referred by patients or their partners^28,29^. COPD diagnosis was oriented by the existence of known risk factors (tobacco smoking, environmental exposure, recurrent acute airways infections in childhood, alpha 1 anti-trypsin deficiency) and of clinical symptoms and signs such as chronic, productive cough, wheezing, dyspnea, chest x-rays supporting the diagnosis. and it was confirmed by pulmonary function tests according to current guidelines^30^. Comorbid diseases were recorded through a systematic and detailed clinical history report. Similarly, data related to tobacco smoking habits were also recorded. All patients were in stable conditions, and free from any acute exacerbations of chronic diseases at the time of the study recruitment.

### Sleep Evaluation

The suspected diagnosis of OSAS was tested through hospital full unattended type 2 PSG (>7 channels) according to current international guidelines^31,32^, using a dedicated device (produced by SOMNOmedics GmbH of Randersacker, Germany).

‘Sleep apnea’ was defined as a complete or almost complete cessation of airflow, indicated by a reduction to 25% or less of baseline amplitude for 10 seconds or more, and ‘hypopnea’ was defined as a clear decrease in airflow to 50% of baseline amplitude for at least 10 seconds. OSAS severity was rated by the frequency of apneic and hypopneic events per hour of sleep (Apnea-Hypopnea Index [AHI]: mild (AHI ≥ 10/h and < 20/h), moderate (AHI ≥ 20/h and < 30/h) and severe (AHI ≥ 30/h)). Hypoxemia in OSAS was defined as an oxygen reduction of over 30% of the total sleep time, with oxy-haemoglobin saturation below 90%^33^.

### Pulmonary Function Tests

Respiratory function tests and blood gas analyses were performed in the morning, participants were fasted and smoke free for at least 12 hours^30^.

Forced expiratory volumes were measured using a water-sealed bell spirometer (Biomedin of Padua, Italy) following acceptability and reproducibility criteria proposed by the American Thoracic Society and by the European Respiratory Society (ATS/ERS)^34^. The maneuver was repeated after salbutamol inhalation and post-bronchodilator data were used to characterize COPD patients^34^. Total Lung Capacity (TLC) and Residual Volume (RV) were obtained using the Helium-rebreathing technique^35^. Values were expressed as a percentage of the predicted value calculated using standardized reference equations^36^. ‘Hypoxia’ constituted an exclusion criterion and was defined as a blood gas analysis with an oxygen partial pressure under 80 mmHg.

### Breath Collection

Since many environmental and metabolic circadian variables may affect exhaled air composition^37^, breath collection was obtained in the morning. Patients were fasted and tobacco smoke free for at least 12 hours. All patients were requested to wash their mouth, without using toothpaste, before the procedure. Similarly, they were not allowed to receive alcoholic or sweetened beverages, or sleep medications before the breath sampling.

A three minute, tidal volume breathing into a dedicated storage device for direct sampling of exhaled breath on adsorbing cartridge (Pneumopipe®, European patent no. 12425057.2, Rome, Italy), was the simple contribution asked of each patient undergone the e-nose test. A detailed description of the study materials and procedure is available elsewhere^38^.

The mouthpieces used for the sampling apparatus were disposable.

### Sensors

The transducers used for the gas sensor array were seven quartz crystals with a resonance frequency of 20 MHz in thickness shear mode, covered with a combination of anthocyanins extracted from three different plant tissues: red rose, red cabbage, blue hortensia. The sensors composing the array were: sensor 1, RR (Red Rose extract 65 mM); sensor 2, BH (Blue Hortensia extract 65 mM); sensor, 3 RC (Red Cabbage extract 65 mM); sensor 4, RRS (Red Rose extract 65 mM + Sucrose 10 mM); sensor 5, RCS (Red Cabbage extract 65 mM + Sucrose 10 mM); sensor 6, BHS (Blue Hortensia extract 65 mM + Sucrose 10 mM); sensor 7, S (Sucrose 10 mM). The electronic board controlling the whole system was based on a STM 32 F303VC, fabricated by ST Microelectronics (Geneva, Switzerland).

A calibration study on this array of biosensors is reported elsewhere^38^. This multisensorial system is able to analyse the volatile and liquid parts of a sample, but for the scope of this work, only the gas sensor array was used. The final dataset is composed of 28 responses resulting from the registration of the 7 sensors behaviour with a sample at four different temperatures (procedure dependent on the exhaled breath sampling on the adsorbent cartridge, explained in detail in the section regarding breath collection and delivery).

### Breath Delivery

The following desorption of the cartridge into the sensors chamber was obtained thanks to an interfacing device able to evenly heat the tube from 50 °C to 200 °C, and finally clean the cartridge keeping the temperature at 300 °C for five minutes. The final fingerprint of the exhaled breath (BreathPrint: BP) was a sequence of four n-dimensional patterns, composed of n responses of n-dimensional gas sensors array at four given temperatures (50–100–150–200 °C).

Individual BPs were graphically represented by radar plots. Each radar plot is made of equi-angular radii, with each radius representing one of the 28 sensor responses. The magnitude of each sensor response is given by the radius length. The radar plot “profile” consists of a line connecting the data values of each radius.

### Analytical Approach

Data are described as mean (and 95% Confidence Intervals, CI) for continuous variables, and as percentage for categorical variables.

A Partial Least Square Discriminant Analysis (PLS-DA) was performed on the 7-dimensional data array, in order to build a model to predict respiratory function parameters. The PLS-DA model was cross-validated through the leave-one-out criterion and then, the Root Mean Square Error in Cross-Validation (RMSECV) of the model in the prediction of respiratory function indexes was computed. The RMSECV provides a measure for the robustness of the PLS-DA model^39^.

In the attempt to exclude the confounding role of tobacco smoking, we repeated the analysis limited to never smokers.

**Figure 1:**
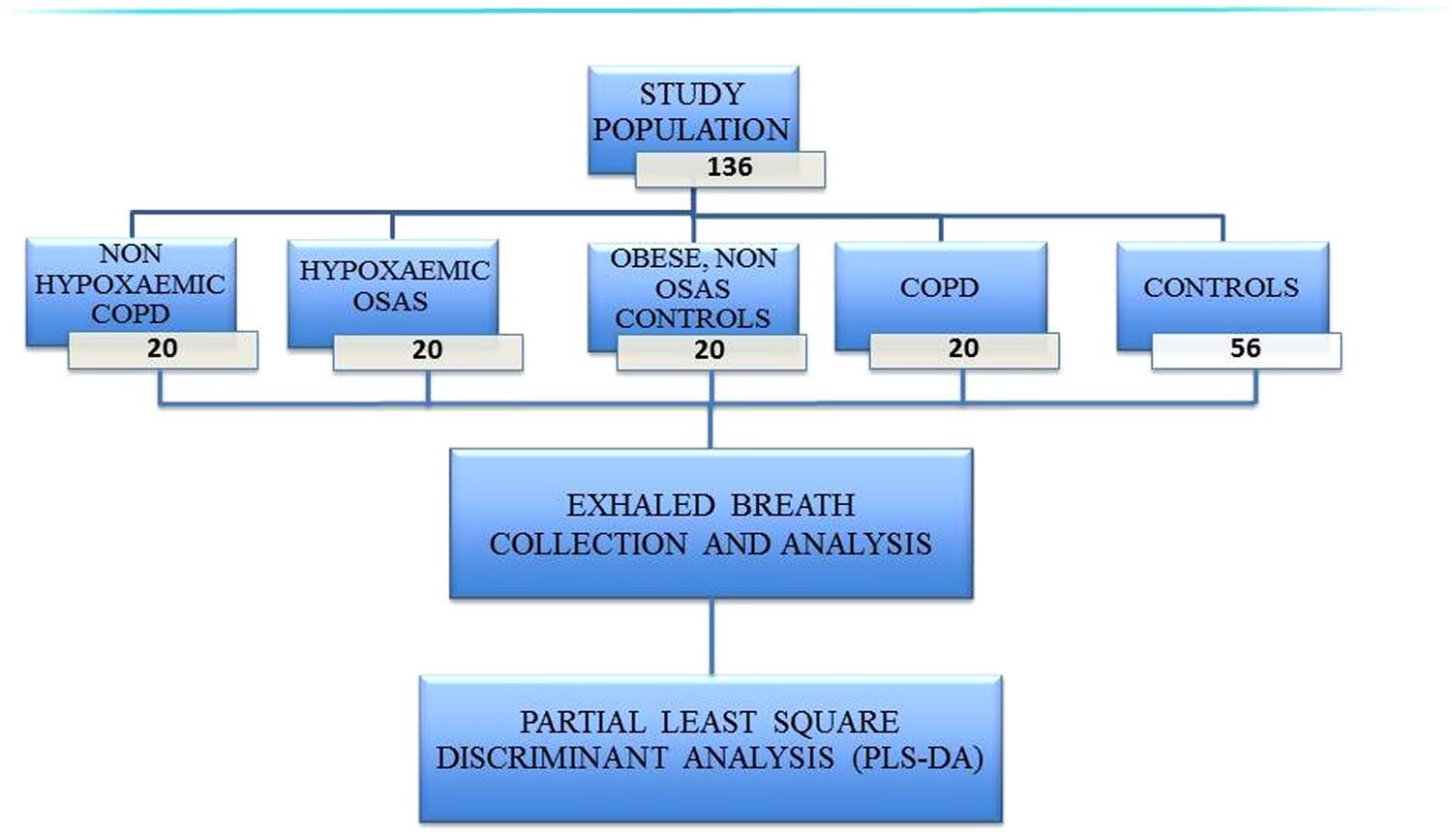
Study design.

A summary flow chart of the study design is displayed in Fig. 1.

### Data availability

The datasets generated and analyzed during the current study are available from the corresponding author on reasonable request.

## Results

Anthropometric and Demographic characteristics, as well as mean numbers and a list of major comorbidities, are included in Table 1. Patients were predominantly males, with a mean age slightly above 60. Groups did not differ in age, sex distribution, number and prevalence of major comorbidities, except for a lower prevalence of hypertension in the COPD group versus the control, obesity and OSAS groups. However, the control and COPD groups had a significantly lower BMI. Moreover, as a consequence of the study design, the mean time with an oxygen saturation below 90% was also higher in the group of hypoxic OSAS.

Finally, the prevalence of current smokers ranged from null in COPD group to 40% in hypoxic OSAS, with figures of 14–15% in controls and hypoxic OSAS and 25% in obese non OSAS groups.

**Figure 2:**
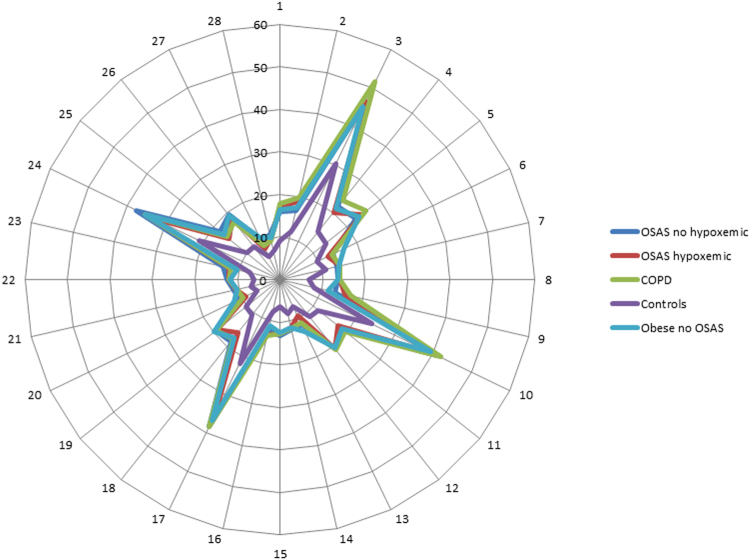
Radar plot comparing profiles of individual groups.

Data displaying e-nose discriminative ability is reported in Table 2. The discriminant analysis substantially failed to correctly differentiate the groups with the only exception of the control group where a 100% correct classification rate was achieved (Table 2). The graphical analysis of radar plots representing the groups BP profiles, clearly confirms the absence of relevant differences in sensor by sensor measures among the groups, except for the distinctive pattern of the control group (Fig. 2).

**Table 2:** Partial least square discriminant analysis of the whole sample with (upper panel) and without smokers (lower panel).

	Obese non-OSAS	Non-hypoxic OSAS	Hypoxic OSAS	COPD	Controls	% of correct classification
Obese non-OSAS (n = 20)	**9**	2	1	5	3	**45**
Non-hypoxic OSAS (n = 20)	1	**12**	1	4	3	**60**
Hypoxic OSAS (n = 20)	5	5	**7**	6	2	**35**
COPD (n = 20)	0	4	1	**12**	3	**60**
Controls (n = 56)	0	0	0	0	**56**	**100**
**Sample w/o smokers = 119**	**Controls**	**Non-hypoxic OSAS**	**Hypoxic OSAS**	**Obese non-OSAS**	**COPD**	**% of correct discrimination**
Controls (n = 56)	**56**	0	0	0	0	**100**
Non-hypoxic OSAS (n = 11)	2	**3**	1	3	2	**27**
Hypoxic OSAS (n = 17)	4	1	**4**	4	4	**23**
Obese non-OSAS (n = 15)	2	0	1	**11**	1	**73**
COPD (n = 20)	3	0	0	4	**13**	**65**

Abbreviations: OSAS = Obstructive Sleep Apnea Syndrome; COPD = Chronic Obstructive Pulmonary Disease.

In the analysis limited to non-smokers (Table 2) the correct classification rate for COPD and obese non-OSAS patients improved, but the correct classification rate for OSAS patients worsened further.

The confounding role of tobacco smoking emerges in the set of multiple comparisons among OSAS, COPD and controls (Table 3), and between OSAS and COPD alone (Table 4), where the rate of correct classification increased after smokers were excluded (Table 4). Interestingly, as already observed in the overall comparison (Table 2), the COPD group showed the largest improvement in classification rating (raising from 40% to 80%), whereas the discrimination of OSAS patients from COPD was excellent even when smokers were considered (92.5% and 92.8%, with and without smokers, respectively) (Table 4).

**Table 3:** Partial least square discriminant analysis among COPD, hypoxic OSAS, non-hypoxic OSAS, and control subgroups in the study sample including (upper panel) and excluding (lower panel) smokers.

	COPD	Non-hypoxic OSAS	Hypoxic OSAS	Controls	% of correct discrimination
COPD (n = 20)	**12**	4	1	3	**60**
Non-hypoxic OSAS (n = 20)	4	**12**	1	3	**60**
Hypoxic OSAS (n = 20)	6	5	7	2	**35**
Controls (n = 56)	0	0	0	**56**	**100**
**Without Smokers**					
COPD (n = 20)	**17**	0	1	2	**85**
Non-hypoxic OSAS (n = 11)	2	**6**	2	1	**30**
Hypoxic OSAS (n = 17)	6	1	**6**	4	**30**
Controls (n = 56)	0	0	0	**56**	**100**

Abbreviations: OSAS = Obstructive Sleep Apnea Syndrome; COPD = Chronic Obstructive Pulmonary Disease.

**Table 4:** Partial least square discriminant analysis among OSAS and COPD with and without smokers (upper panels), and after separating OSAS according to the occurrence of hypoxemia (lower panel).

Total sample	All OSAS	COPD	% of correct classification	Without smokers	All OSAS	COPD	% of correct classification
**All OSAS** (**n = 40**)	**37**	3	**92**	**All OSAS** (**n = 28**)	**26**	2	**93**
**COPD** (**n = 20**)	12	**8**	**40**	**COPD** (**n = 20**)	4	**16**	**80**
*w/o smokers	**Hypoxic OSAS**	**Non-hypoxic OSAS**	**COPD**	**% of correct classification**			
**Hypoxic OSAS** (**n = 11**)	**6**	4	1	**54**			
**Non-hypoxic OSAS** (**n = 17**)	0	**13**	4	**76**			
**COPD** (**n = 20**)	3	6	**11**	**55**			

Abbreviations: OSAS = Obstructive Sleep Apnea Syndrome; COPD = Chronic Obstructive Pulmonary Disease.

*In this comparison, the original PLS-DA model was not solid enough to univocally classify all the patients belonging to the Hypoxic, Non-hypoxic OSAS and COPD groups. The reported model was therefore generated after excluding smokers.

Table 5 shows the results obtained by a discriminant analysis limiting the number of groups undergoing comparison to two. A very high classificatory capacity is observed, both between the whole OSAS group and the control group (correct classification rate of 100% and 98%, respectively) (Table 5) and within the OSAS group itself (hypoxic vs non-hypoxic) (correct classification rate of 80% and 60%, respectively) (Table 5). A 100% correct classification was finally obtained when control and COPD groups were compared (Table 5). Interestingly, the classification of COPD patients worsened if hypoxemic and non-hypoxemic OSAS groups were merged (Table 4). This suggests that the discriminatory capacity of VOCs vs COPD is enhanced by the variety of potential allocations.

**Table 5:** Partial least square discriminant analysis among OSAS and Controls (upper panel), between Hypoxic and Non-hypoxic OSAS (middle panel), and between Controls and COPD (lower panel).

	**All OSAS**	**Controls**	**% of correct classification**
All OSAS (n = 40)	**40**	0	**100**
Controls (n = 56)	1	**55**	**98**
	**Non-hypoxic OSAS**	**Hypoxic OSAS**	**% of correct classification**
Non-hypoxic OSAS (n = 20)	**16**	4	**80**
Hypoxic OSAS (n = 20)	8	**12**	**60**
	**Controls**	**COPD**	**% of correct classification**
Controls (n = 56)	**56**	0	**100**
COPD (n = 20)	0	**20**	**100**

Abbreviations: OSAS = Obstructive Sleep Apnea Syndrome; COPD = Chronic Obstructive Pulmonary Disease.

## Discussion

The main finding of this study is that the electronic nose can optimally identify healthy subjects and distinguish them from both OSAS and COPD patients. On the contrary, it substantially failed to discriminate OSAS patients from other groups, but resulted fairly able to discriminate COPD patients and hypoxemic OSAS patients. Thus, the e-nose can identify people unlikely to benefit from PSG, but it cannot distinguish OSAS from obese and COPD patients. Accordingly, it might limit the access to PSG only to some extent. However, even a small preselection is likely to improve the resource allocation, given that OSAS is highly prevalent and PSG is requested more and more frequently. In fact, excluding healthy subjects from PSG screening would improve the cost/efficacy of PSG. Finally, as requested of an ideal screening test, breath analysis is inexpensive, non-invasive, and easy and quick to use.

The great heterogeneity of OSAS likely explains the partly negative conclusions of this study. OSAS heterogeneity was related to multiple aspects, i.e. anatomical or non-anatomical predisposing conditions, pathogenetic mechanisms^40^ and age-dependent phenotypic variability^41^. Several phenotypes may in fact be inferred from PSG features in mild to moderate OSAS^42,43^. Finally, Vavougios and co-workers recently established a clustering among six different comorbidities and OSAS^44^ and, in another recent paper, the cause-effect relationship between comorbidities and BP heterogeneity in OSAS was clearly shown^20^. However, the fact that in our patients the e-nose could better discriminate hypoxemic than non-hypoxemic OSAS patients suggests that the metabolic impact of hypoxemia increases BP specificity.

Our study confirms that COPD represents a promising model for the e-nose analysis^45,46^. Previous VOCs studies could effectively distinguish COPD from normal subjects either through e nose or gas characterizing methods^47–49^. The fact that COPD patient classification worsened if hypoxemic and non hypoxemic OSAS groups were merged, supports the dominant view of COPD as a highly heterogeneous condition (Table 3). In fact, COPD is characterized by systemic and metabolic effects, such as systemic inflammation and oxidative stress, and associated with cardiovascular and metabolic disorders^50–53^. Interestingly, the level of “systemic” inflammation in COPD strictly parallels that of “local” bronchial inflammation and obstruction (FEV1)^51^. Furthermore, pulmonary oxidative stress markers spilling over into mainstream, such as the excretion of F2-isoprostanes, stable products of peroxidation of arachidonic acid^54^, increase in COPD regardless of smoking habits and peak during exacerbations^54^. Thus, a vast array of VOCs variously contributes to shape the BP of COPD and likely make it quite heterogeneous.

To our knowledge, this is the first study testing the discriminative properties of BP obtained through e-nose analysis in a multiple comparison study (Hypoxic and non-Hypoxic OSAS, Non-Hypoxic COPD, Obese non-OSAS) on respiratory patients and healthy controls. Previously published studies, in fact, compared a single pathologic group with controls (i.e. Asthma, COPD, Sarcoidosis, Lung Cancer, OSAS alone vs controls)^41,42,44–46^ or two, rarely three, distinct respiratory disease groups versus each other (i.e, Asthma vs COPD, Malignant Mesothelioma vs Benign Pleural Disease vs Controls)^55,56^. Only Dragonieri *et al*. compared the BP of OSAS, obese non-OSAS and non-obese, non-OSAS subjects^24^. Our findings confirm, through a larger and more articulated comparative analysis, that BP of OSAS and obese patients overlap considerably. Furthermore, in previous studies, the effects of age, gender, tobacco smoking habits and comorbidities were rarely taken into account^20^, and the few existing evidences are limited to exhaled nitric oxide only^57,58^. In our study, groups showed comparable demographic characteristics and comorbidities, and this allowed explore the effects of current smoking habits on BP. Indeed, cigarette smoking qualified as an important confounder, and this likely explains why, at variance with findings recently reported by Dragonieri *et al*.^23^ which pertain to a non smoking population, COPD was frequently misdiagnosed in the OSAS vs COPD comparison (40% correct classification rate only - Table 4).

Limitations of this study deserve consideration. Firstly, we had no direct measure of relevant potential correlates for VOCs prints, such as bronchial inflammation, nitric oxide concentration, etc. Secondly, we recorded VOCs fingerprints, but we could not identify their components through dedicated analyses, such as gas chromatography or mass spectrography. Thus, we can conceivably conclude that VOCs significantly distinguish healthy subjects from a pool of people with chronic respiratory conditions, but we ignore which exhaled molecules account for this distinction. However, the objective of the study was to assess the whole breath pattern and not to characterize it analytically. Finally, our data need confirmation by an external validation set^59^.

In conclusion, our e-nose technology applied to breath analysis allows identify healthy subjects and subsequently to exclude them very reliably from PSG screening, but it cannot recognize OSAS-specific BP. On the other hand, its classificatory and discriminatory properties seem worthy of further testing on people with hypoxemic OSAS and on people with COPD.
